# Enhanced Hydrogen Peroxide Decomposition in a Continuous-Flow Reactor over Immobilized Catalase with PAES-C

**DOI:** 10.3390/polym16131762

**Published:** 2024-06-21

**Authors:** Yunrui Li, Yu Zhang, Wenyu Zhang, Hao Wu, Shaoyin Zhang

**Affiliations:** College of Light Industry and Chemical Engineering, Dalian Polytechnic University, Dalian 116034, China; 210120856000122@xy.dlpu.edu.cn (Y.L.); 210120856000132@xy.dlpu.edu.cn (Y.Z.); 210110703000008@xy.dlpu.edu.cn (W.Z.); 210120857000148@xy.dlpu.edu.cn (H.W.)

**Keywords:** PAES-C, polyaryl ether sulfone, immobilized catalase, continuous flow reactor, hydrogen peroxide decomposition

## Abstract

Due to the specificity, high efficiency, and gentleness of enzyme catalysis, the industrial utilization of enzymes has attracted more and more attention. Immobilized enzymes can be recovered/recycled easily compared to their free forms. The primary benefit of immobilization is protection of the enzymes from harsh environmental conditions (e.g., elevated temperatures, extreme pH values, etc.). In this paper, catalase was successfully immobilized in a poly(aryl ether sulfone) carrier (PAES-C) with tunable pore structure as well as carboxylic acid side chains. Moreover, immobilization factors like temperature, time, and free-enzyme dosage were optimized to maximize the value of the carrier and enzyme. Compared with free enzyme, the immobilized-enzyme exhibited higher enzymatic activity (188.75 U g^−1^, at 30 °C and pH 7) and better thermal stability (at 60 °C). The adsorption capacity of enzyme protein per unit mass carrier was 4.685 mg. Hydrogen peroxide decomposition carried out in a continuous-flow reactor was selected as a model reaction to investigate the performance of immobilized catalase. Immobilized-enzymes showed a higher conversion rate (90% at 8 mL/min, 1 h and 0.2 g) compared to intermittent operation. In addition, PAES-C has been synthesized using dichlorodiphenyl sulfone and the renewable resource bisphenolic acid, which meets the requirements of green chemistry. These results suggest that PAES-C as a carrier for immobilized catalase could improve the catalytic activity and stability of catalase, simplify the separation of enzymes, and exhibit good stability and reusability.

## 1. Introduction

Hydrogen peroxide is widely used as a disinfectant for germs, but its residue may cause harm to the environment and the human body. Catalase is a biocatalyst that catalyzes hydrogen peroxide specifically; it not only can remove residual hydrogen peroxide, but also catalyze the products generated by the reaction of water and oxygen, which makes its use environmentally friendly [1,2,3]. Catalase has wide applications in the food, papermaking, medicine, textile, and other industries [4,5,6,7]. In industry, in the free state, catalase cannot be recycled due to unsatisfactory stability, greatly reducing its practical applications [8,9,10]. Thus, research on immobilized catalase is of great significance for environmental protection. In view of the shortcomings of free catalase in industrial applications, immobilized-catalase technology came into being [11,12,13,14,15,16,17]. The choice of vector is critical because biocatalysts will exhibit excellent mechanical, chemical, biochemical, and specific kinetic properties based on the interaction of the vector with the enzyme [18,19,20,21,22,23]. At present, the selection and manufacture of suitable enzyme-immobilization vectors and the design and optimization of immobilized-enzyme methods with high catalytic activity have become research hotspots in immobilized-enzyme technology [24,25,26].

The main immobilization methods for CAT (catalase) are adsorption, crosslinking, covalent binding, etc. Among them, adsorption is the simplest, and the economic value is relatively high, but the operational stability is low, which is not conducive to industrialization [27]. Covalent binding results in long-lasting enzymes and high enzyme recoveries, making them reusable, and helps to enhance the stereoselectivity and stability of enzymes [28]. However, covalent binding requires relatively complex steps and long incubation times for proper fixation and therefore has limitations [29]. The method of glutaraldehyde cross-linking fixation will destroy the active center of the enzyme and greatly reduce the activity of the enzyme [30]. Encapsulation-and-fixation techniques involve the immobilization of multiple biomolecules, including sensitive enzymes and other cellular content, in tiny vesicles with porous membranes [31]. The immobilized catalase prepared by Yulin Wang et al. with gelatin-encapsulated activated carbon as the carrier accounted for 65.69% of the natural enzyme activity, and its residual activity was still as high as 80% after 15 uses [32]. However, there are some limitations to this technique. For example, diffusion is a serious problem, and if the reaction products accumulate rapidly, it can lead to membrane rupture [33]. In a paper by Evren Sel et al., the CAT enzyme was immobilized on the P(MMA-co-PEG500MA) copolymer by a physisorption method. A high immobilization yield (76%) was achieved under optimized conditions. The immobilized enzyme displayed improved tolerance towards pH and temperature changes [34]. Alptekin Özlem et al. covalently immobilized bovine liver CAT on Eupergit C, and the resulting immobilized catalase had good reusability, thermal stability. and long-term-storage stability [35]. Domink L. Jürgen-Lohmann et al. used a sol-gel approach to fix the CAT, and the immobilized enzyme remained stable in 17 consecutive reactor batch cycles with no apparent loss in activity [27]. Gold (AuNPs) and silver (AgNPs) nanoparticles have been prepared by the “one-pot” synthetic method in the presence of poly(N-vinylpyrrolidone) (PVP), and they were loaded onto the ZnO surface by the impregnation method. The decomposition of H_2_O_2_ was carried out by supramolecular catalysis simulating CAT, and it was found that its recyclability was good [36]. Jyoti Kaushal et. used chitosan and chitosan-bentonite as the carrier and CAT as the enzyme source and fixed the CAT, and the enzyme activity was 50% and 70% of the initial enzyme activity after 20 cycles [37].

As immobilized-enzyme carriers, polymer materials have the advantages of low cost and a wide range of sources [38,39]. The synthesis of polymeric structures that can be used in enzyme-immobilization applications is seen as an important target for today’s technology [40]. Due to its excellent mechanical properties, acid and alkali resistance, solvent resistance, good thermal stability, porosity, and poor hydrolysis [41,42], polyaryl ether sulfone can be used in ultrafiltration membranes [43] and adsorbents for heavy metals [44]. Moreover, polyaryl ether sulfone support is prepared from the carboxylic-group-containing renewable resource bisphenolic acid, which not only has low cost and easy separation, but also has good adsorption between functional carboxyl groups and inactive sites of enzymes. Therefore, polyaryl ether sulfone as a carrier for immobilized enzymes not only provides physical protection for the enzyme, but also maintains a certain degree of freedom for the enzyme inside.

Based on the above considerations, the modified PAES-C-immobilized catalase was selected in this work, and a continuous-operation device that is conducive to large-scale production, convenient recovery and easy industrial use was designed to catalyze the decomposition of hydrogen peroxide.

## 2. Materials and Methods

### 2.1. Materials

4,4’-Dichlorodiphenylsulfone (DCDPS) and bisphenolic acid (DPA) were purchased from McLean Biochemical Technology Co. (Shanghai, China). Tetrahydrofuran (THF), anhydrous potassium carbonate (K_2_CO_3_), copper sulfate pentahydrate, and N,N-dimethylacetamide (DMAC) were purchased from Sinopharm Chemical Reagent Co. (Shanghai, China). Anhydrous ethanol, toluene, potassium hydroxide, dimethyl sulfoxide (DMSO), disodium hydrogen phosphate (Na_2_HPO_4_), sodium dihydrogen phosphate (NaH_2_PO_4_), K90, cross-linked PVP, and hydrochloric acid 37% were purchased from Kemi Chemical Reagent Development Center (Tianjin, China). Catalase was purchased from Fengtai Co. (Shandong, China). Hydrogen peroxide (H_2_O_2_) was purchased from Dongfang Guangcheng Pharmaceutical Chemical Co. (Tianjin, China). The purity of the above chemicals was analytical-grade.

### 2.2. Experimental Process

#### 2.2.1. Synthesis of Polyaryl Ether Sulfone Containing Carboxyl Side Chain

Firstly, the reactants were added to the three-ported flask that had been connected to the water separator in the ratio n_DCDPS_:n_DPA_:n_KOH_:n_K2CO3_ = 1:1:1:1:1.2, and then the solvent (4 A molecular sieve immersed for 48 h) was added in accordance at a ratio of V_Toluene_:V_DMAC_ = 2:3.

The flask was connected with nitrogen and water separators from left to right and was heated to 110 °C for 2 h after the temperature had been raised to reflux; it then continued to be slowly heated up to 140–150 °C over 3 h. Finally, the temperature was raised to 160 °C and held constant for 24 h until the products in the reaction system climbed the pole and solid-liquid separation indicated the end of the reaction. The entire heating reaction process was conducted under nitrogen protection and mechanical stirring. At the end of the reaction, the solid product was transferred to a conical flask and a mixed solution of V_THF_:V_HCl_ = 4:5 was added for acidification by immersion. After immersion for 24 h, the solid product was washed with water and ethanol in five repeated cycles of boiling to obtain a white solid product, and the product was dried in a vacuum oven at 100 °C for 24 h to obtain the target product, polyaryl ether sulfone, which contains carboxyl side chains (PAES-C).

#### 2.2.2. Preparation of Carrier

Michler Andre et al. investigated the effect of PVP on the enzymatic activity of CAT; they found that the binding of PVP to CAT had no negative effect on the catalytic activity of the enzyme. They further found that if the polymer binds to CAT, the enzymatic activity of CAT increases. Additionally, they found that the structure of CAT changes after binding so the active site is exposed, and it was also determined that CAT can decompose H_2_O_2_ in the presence of PVP [45]. With the increase in the relative molecular weight of PVP, the pore of the prepared carrier gradually increased, and the adsorption rate and recovery rate of the enzyme also increased gradually.

Due to the different structures of PVP and PVP-P, which may lead to different enzyme activities, two doping methods, namely 10% PVP K90 and 10% PVP-P (cross-linked PVP), were selected to make porous supports, and changes in their performance with immobilized enzymes was investigated.

K90 and PAES-C with mass fractions of 12, 15, 18, and 20% were added to a certain volume of dimethyl sulfoxide (DMSO) solution, K90 (see Table 1), and the mixed solution was dropped into deionized water with a glass dropper to make it spherical. The white solid was filtered and boiled 4–5 times in ionized water, then vacuum-dried at 100 °C for 24 h to obtain K90-doped carriers (PAES-C/K90). PVP-P-doped carriers (PAES-C/P) were prepared using the same method.

#### 2.2.3. Preparation of Immobilized Catalase

To prepare the immobilized catalase, 0.05 g (dry weight) of PAES-C/K90 and PAES-C/P were added to the same enzyme solution prepared with 0.2 m mol·L^−1^ phosphate buffer solution and the mixture was shaken at an appropriate temperature and speed on a shaker for a certain period of time. The supernatant was stored to calculate the immobilization efficiency, and then washed three times with buffer to complete the immobilization of CAT and prepare immobilized catalase (PAES-C-CAT). The product was allowed to dry at room temperature and stored in the refrigerator at 4 °C for later use.

#### 2.2.4. Enzyme Activity of Free and Immobilized Enzyme

Free-enzyme activity (See Supplementary Material Formula (S1) for calculation formula) was determined spectrophotometrically by measuring the decrease in H_2_O_2_ absorbance at 240 nm (See Supplementary Material Figure S1 for the relationship between absorbance and substrate concentration) in a reaction mixture containing 2.9 mL of substrate (pH: 7, 0.1 M H_2_O_2_) and 0.1 mL of CAT (1 mg/mL). The reaction mixture was maintained at 25 °C for 2 min, and the enzymatic reaction was stopped by adding 0.5 mL of 1 M HCl. For immobilized-enzyme activity (See Supplementary Material Formula (S2) for calculation formula), copolymeric samples (0.05 g) were mixed with the substrate solution prepared as above at 25 °C for 2 min. After 2 min, the samples were removed and the reaction was stopped by adding 0.5 mL of 1 M HCl. One unit (U) of enzyme activity was defined as the quantity of enzyme catalyzing the decomposition of 1 µmol H_2_O_2_ per min under optimum assay conditions. The maximum activity in all enzyme results was assumed to be 100%, and the results were given as relative activity. In addition, all enzyme-activity measurements were carried out using three parallel samples, and standard deviations were added to the corresponding graphs.

#### 2.2.5. Selection of Cu^2+^ Concentration

H Sigel et al. found that Cu^2+^ can act as a “molecular probe” between the structure of the complex and the activity of catalyzing the decomposition of H_2_O_2_, as it subtracts the coordination group in the complex from its catalytic activity. Additionally, since the ligand group is oxidized by H_2_O_2_, the ligand information of the metal ion can be further oxidized from some parts of the ligand, thereby increasing the catalytic activity of CAT [46]. Alexander S. Lukatkin found that Cu^2+^ ions had a greater effect on oxidative stress in cucumber seedlings than in cucumber callus (i.e., more negative). Catalase (CAT) and ascorbate peroxidase (APOX) activity are both increased in calli and plantlets, but the increased levels of these antioxidant enzyme systems depend on Cu^2+^ concentrations [47]. Helmut Sigel et al. confirmed that Cu^2+^ in aqueous solution can accelerate the rate of H_2_O_2_ decomposition. The reaction mechanism may be outlined as follows [48]:

(1) $${\mathrm{Cu}}^{2+}+H_{2}O_{2}⇌{\mathrm{Cu}(H_{2}O_{2})}^{2+}$$

(2) $${\mathrm{Cu}(H_{2}O_{2})}^{2+}⇌{\mathrm{Cu}(\mathrm{OOH})}^{+}+{\;H}^{+}$$

(3) $${\mathrm{Cu}(\mathrm{OOH})}^{+}+H_{2}O_{2}⇌\mathrm{Cu}(\mathrm{OOH}){\left(H_{2}O_{2}\right)}^{+}$$

(4) $$\mathrm{Cu}\left(\mathrm{OOH}\right){\left(H_{2}O_{2}\right)}^{+}⟶{\mathrm{Cu}}^{2+}+O_{2}+H_{2}O+{\mathrm{OH}}^{-}$$

In order to explore the effect of Cu^2+^ on the activity of immobilized enzymes, CuSO_4_·5H_2_O was prepared in Cu^2+^ solutions with different concentrations and the pH was adjusted to 7. Next, 0.5 g of PAES-C/P was soaked in different concentrations of ionic solution for 14 h, the catalase was fixed according to the above method, a porous carrier for the adsorption of Cu^2+^ was fabricated (immobilized catalase), and the immobilized-enzyme activity after treatment was determined. A blank sample was made with no Cu^2+^ solution carrier.

#### 2.2.6. Immobilized Enzyme Single-Factor Assay

The effects of different enzyme concentrations (16, 32, 48, 64, 80, 96, 112 U/mL), different immobilization times (2, 4, 6, 8, 10, 12 h), and different immobilization temperatures (25, 30, 35, 40, 45, 50 °C) on the activities of immobilized enzymes were studied by setting the carrier amount to 0.05 g, Cu^2+^ concentration to 3 mmol/L (see Table 2), shaking speed to 150 r/min, and pH value to 7.0.

#### 2.2.7. Model of the Continuous Experimental Device

**Figure 1:**
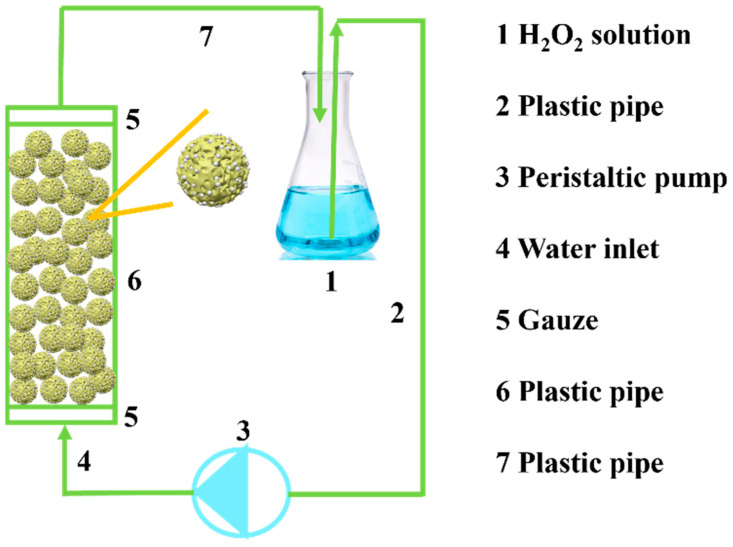
Continuous experimental device model.

The model of the continuous device used in this experiment is shown in Figure 1. The inner diameter of the plastic pipe is 1 cm, and it is about 10 cm long. The gauze is fixed at both ends, the immobilized enzyme is used as the reactor, and the flow rate of the peristaltic pump is regulated according to the conditions.

#### 2.2.8. Continuous Catalytic H_2_O_2_ Decomposition Tests

Under the action of peristaltic pump 3 (in which vacuum and pressure are created by compressing and releasing the plastic tube, which enables the transport of liquids), the H_2_O_2_ solution enters the reactor through plastic pipe 2 through inlet 4 and flows out from outlet 7 after the reaction. The rate of decomposition of hydrogen peroxide by immobilized enzymes under different initial concentrations (0.035, 0.045 mol/L), flow rates (4, 8, 12 mL/min) and different masses of immobilized enzyme (0.1, 0.15, 0.2 g) was investigated (see Table 3). Samples were taken at one point at 5 min intervals, and the H_2_O_2_ concentration was measured with a UV spectrophotometer.

### 2.3. Characterization

#### 2.3.1. Fourier-Transform Infrared Spectra (FT-IR)

The Fourier-translation infrared spectra (FT-IR) was recorded on a FT-IR spectrometer (Nicolet, Nicolet IS50) (Waltham, MA, USA). The sample was first pretreated and tableted before measurement. A small amount of sample was evenly mixed with a certain amount of dried KBr powder, ground, and pressed into a transparent sheet (2 MPa) on the tablet press. The infrared spectrum scanning range was 400~4000 cm^−1^.

#### 2.3.2. Scanning Electron Microscope (SEM)

An SM-7800F scanning electron microscope (SEM) manufactured by Japanese Electronics Company (Shibuya-ku, Tokyo, Japan) was used to analyze the morphology and morphology of the materials. Samples were glued to the prepared conductive adhesive, and the samples with unglued surfaces were removed by high-pressure spray gun. After that, the samples were gold-plated in vacuum and placed in a vacuum mirror barrel for observation by SEM.

#### 2.3.3. ^1^H-NMR

The molecular structure of the synthesized polymer was probed by 1H-NMR on a Varian INOVA 400 MHz nuclear magnetic resonance apparatus (Santa Clara, CA, USA). DMSO-d6 was selected as the solvent with TMS as the internal standard.

#### 2.3.4. Gel Permeation Chromatograpgy (GPC)

The molecular weights (Mw) and polydispersity index (PDI = Mw/Mn) were measured by gel-permeation chromatography (GPC, Waters 2414) (Milford, MA, USA) with a Styrogel@ column. During the measurement, 0.5 mL/min chromatographically pure DMSO was used as the eluent. The molecular weight of Pluslan polysaccharide was calibrated with a range of 10^3^~10^6^, and its relative molecular weight was tested.

#### 2.3.5. UV-Vis Spectrophotometry

The change in absorbance of catalyzed hydrogen peroxide catalyzed by CAT was examined on a UH 5300 UV-Vis spectrophotometer (HITACHI, Tokyo, Japan) at the characteristic absorption peak of 240 nm.

### 2.4. Stability of Catalase Immobilized on PAES-C

#### 2.4.1. pH Stability

The effects of pH on the activities of free and immobilized catalase were studied by using 0.2 M phosphate-buffered saline solution (pH 5.0, 6.0, 7.0, 8.0, 9.0) as raw material and stored at different pH values for 5 h. The initial activities of both samples were considered 100%.

#### 2.4.2. Thermal Stability

The effects of temperature on the activities of free and immobilized catalase were studied by placing free and immobilized catalase in phosphate buffer solution at pH 7.0 and stored at 40, 50 and 60 °C for 30, 60, 90 and 120 min respectively. The initial activities of both samples were considered 100%.

#### 2.4.3. Storage Stability

The prepared immobilized enzymes were placed in air and stored at 4 and 25 °C for 28 days. Their enzyme activities were calculated every seven days, and the storage stability of the free enzymes was determined as a control under the same conditions. The initial activities of both samples were considered 100%.

#### 2.4.4. Operational Stability

The reusability of the immobilized catalase was also investigated. The immobilized catalase was exposed to fresh H_2_O_2_ solution, and the activity was measured according to assay activity. After the immobilized catalase was washed, it was exposed to the same concentration of substrate again. This procedure was repeated 22 times, and the enzyme activity was measured after each step. The activity of the enzyme after the first use was considered 100%.

## 3. Results

### 3.1. Characterization of PAES-C

**Figure 2:**
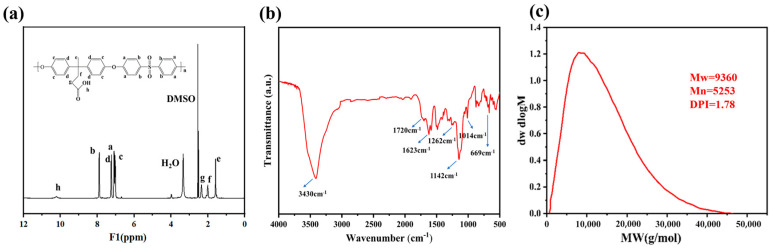
(**a**) ^1^H-NMR spectrum of PAES-C, (**b**) FT-IR spectrum of PAES-C, (**c**) Molecular weight distribution of PAES-C.

As can be seen from the infrared spectra of Figure 2a, there are absorption peaks generated by the stretching vibrations of −OH and C=O in the carboxyl group at 3430 and 1720 cm^−1^, and the above two characteristic peaks prove that there is a carboxyl group on the polymer; absorption peaks generated by the stretching vibration of the ether bond between the benzene rings are found at 1248 and 1014 cm^−1^, and it can be proved that the ether bond is formed between the molecules of the polymer at the same time. At the same time, absorption peaks generated by −SO_2_− can be found at 1262 and 1142 cm^−1^, and an infrared characteristic absorption peak generated by the stretching vibration of the −C-S− bond exists at 669 cm^−1^. The above characteristic peaks indicate that DPA and DCDPS successfully reacted.

From the ^1^H-NMR spectrum of Figure 2b, it can be seen that there are chemical shifts of proton hydrogens on the long chain of the aliphatic polymer molecule in the range of 1.5 ppm to 2.5 ppm at g, e, and f. The peaks of a, b, c, and d are the chemical shifts of proton hydrogens on the aromatic ring. Meanwhile, the chemical shift generated by H on −COOH appears at 10.5 ppm. The experimental results can prove that the synthesis of poly(aryl ether sulfone) containing a carboxyl side chain was achieved.

As shown in Figure 2c, the average molecular weight of PAES-C was 9360 g/mol, and the polydispersity index (DPI) was 1.78. Overall, the distribution of the molecular weight of the polymer is relatively uniform.

**Figure 3:**
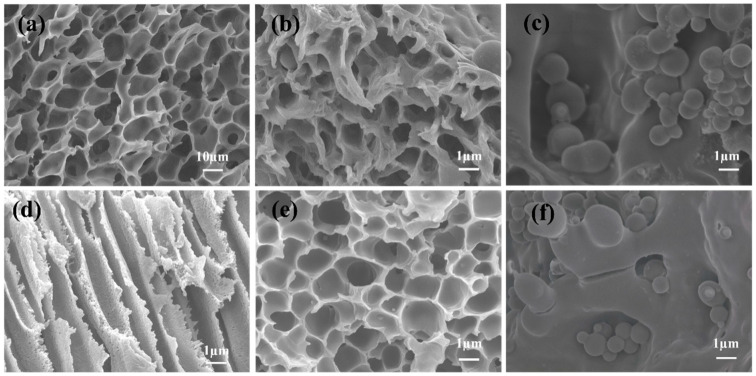
(**a**) Frontal SEM image of PAES-C, (**b**) SEM image of PAES-C/K90, (**c**) SEM image after immobilization of CAT in PAES-C/K90, (**d**) Side SEM image of PAES-C, (**e**) SEM image of PAES-C/P, (**f**) SEM image after immobilization of CAT in PAES-C/P.

As can be seen in Figure 3a,d, the front and side of PAES-C presents a pore structure, and the formation of pores is directly related to the carboxyl side chain of PAES-C. The porous structure increases its specific surface area and roughness so that it has more adsorption sites and spatial sites and has better adsorption performance. However, the pore size did not meet the requirements of CAT for immobilizing carriers in this experiment (the pore size is too small for encapsulation of catalase), and the distribution of pores was also disorderly, which was not conducive to the subsequent adsorption of enzymes and the exertion of enzyme activity. Juan M. Bolivar et al. compared three internally ordered and two amorphous silica materials as enzyme carriers (pore size ≥ 10 nm per material) for the coimmobilization of D-amino acid oxidase and CAT and found that the uniform pore structure framework was beneficial to enzyme loading, immobilization rate, and catalytic efficiency, and the results confirmed that the internal structure could be adjusted to optimize the vector of enzyme activity and performance [49]. The surface of the carrier after immobilization of the enzyme was analyzed and compared with that before immobilization of the enzyme, and the comparison graphs are shown in Figure 3b,c,e,f. PAES-C/K90 has an irregular porous structure, and after the immobilization of the enzyme, globules could be observed on the surface of the carrier and some holes were filled. The pores of PAES-C/P are uniform and dense, and after the immobilization of enzyme, the spheroids filled the pores in large quantities and there was a large amount of accumulation and attachment on the surface.

**Figure 4:**
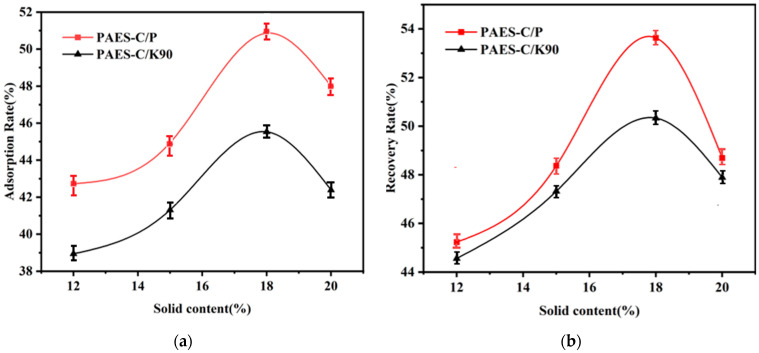
(**a**) Enzyme adsorption rates of vectors with different mass fractions, (**b**) Enzyme recovery of vectors with different mass fractions.

The adsorption rate (See Supplementary Material Formula (S3) for calculation formula) and recovery rate (See Supplementary Material Formula (S4) for calculation formula) of PAES-C direct immobilization catalase were 37% and 21%, respectively. PAES-C doping with K90 or PVP-P can effectively control the pore size and pore distribution of PAES-C, aiming to improve its ability to immobilize enzymes. Both the adsorption rates and recovery rates of the immobilized-enzyme are shown in Figure 4a,b. When the solid content is 18%, the adsorption rate and recovery rate of PAES-C/K90 and PAES-C/P reach the maximum. The adsorption rate and recovery rate of PAES-C/K90 are 46 and 50%, and the adsorption rate and recovery rate of PAES-C/P are 51 and 54%. It can be seen that the adsorption rate and recovery rate of PAES-C/P are higher than those of PAES-C/K90 under the condition of the same amount of free enzyme, the dense pore structure of PAES-C/P can provide more adsorption sites for catalase. The above results show that PAES-C doping with K90 or PVP-P can effectively control the pore size and pore-size distribution of PAES-C and improve its ability to immobilize enzymes.

### 3.2. Immobilized Catalase Condition Optimization

**Figure 5:**
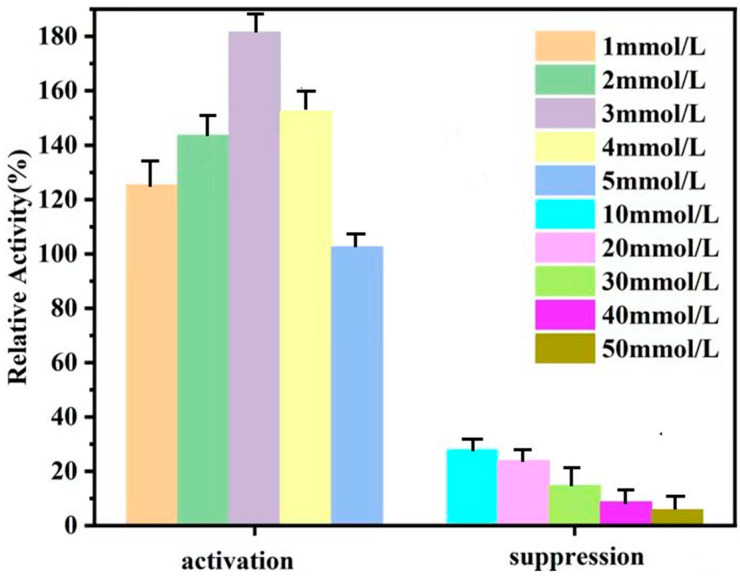
Effect of different concentrations of Cu^2+^ on immobilized-enzyme activity.

CAT was immobilized by impregnating treated PAES-C/P with different concentrations of Cu^2+^ to explore the effect of Cu^2+^ on immobilized-enzyme activity, as shown in Table 4 and Figure 5. Different Cu^2+^ concentrations activate (inhibit) immobilized-enzyme activity. A Cu^2+^ concentration greater than 10 mmol·L^−1^ inhibits immobilized-enzyme activity, possibly because Cu^2+^ preempts the catalase site, resulting in a large amount of catalase shedding. A concentration of Cu^2+^ of 3 mmol·L^−1^ had the best effect on the activation of immobilized-enzymes, with a relative enzyme activity of 182%. From the above research and experimental conclusions, it is found that appropriate amounts of Cu^2+^ can promote the activity of CAT and improve the catalytic efficiency of enzymes, so 3 mmol·L^−1^ Cu^2+^ was selected as the activator of immobilized enzymes in order to maximize the activity of the enzymes.

**Figure 6:**
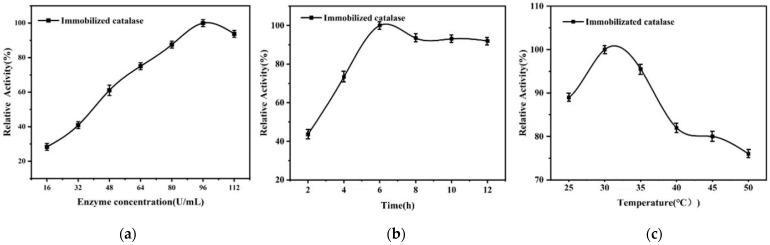
(**a**) Effect of free-enzyme concentration on immobilized-enzyme activity, (**b**) Effect of immobilization time on immobilized-enzyme activity, (**c**) Effect of immobilization temperature on immobilized-enzyme activity.

In the CAT-immobilization experiment, the immobilization experimental conditions were selected to maximize the activity of the enzyme. A single-factor experiment was performed using PAES-C/P as the carrier, including the amount of free-enzyme solution, the time of immobilization of the enzyme, and the temperature. The results are shown in Figure 6a–c. The concentration of free enzyme was changed sequentially over a gradient of 16 U/mL. The immobilized-enzyme activity gradually increased with the increase in free-enzyme dosage, and the relative enzyme activity of the immobilized enzyme reached a maximum at 96 U/mL. With the change in immobilization time, the activity of the immobilized enzyme showed a trend of first increasing and then decreasing, and at 6 h, the relative enzyme activity reached the maximum. The ambient temperature increased from 25 °C to 50 °C, and the relative enzyme activity of the immobilized enzyme was like a mountain peak: at a temperature of 30 °C, relative enzyme activity peaked, after which it decreased. This shows that the above three factors have different degrees of influence on the immobilization of enzymes. The optimal environmental conditions for immobilized enzymes are free-enzyme dosage of 3 mL, immobilization time of 6 h, and temperature of 30 °C.

### 3.3. Stability of Catalase Immobilized on PAES-C

**Figure 7:**
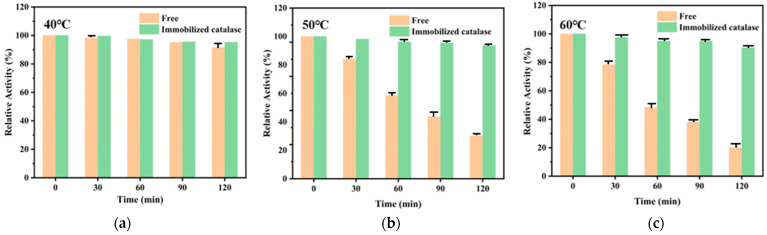
Comparison of the thermal stability of immobilized enzyme and free enzyme at 40 °C (**a**), 50 °C (**b**) and 60 °C (**c**).

A comparison of enzyme activity after the same retention time of immobilized enzyme and free enzyme at the three different temperatures investigated is shown in Figure 7a–c. When the temperature was 40 °C, the free enzyme maintained more than 90% activity after 2 h storage, while the immobilized enzyme maintained more than 95% activity. When the temperature was increased from 40 °C to 50 °C, the enzyme activity of free enzyme after storage for one hour was 59% of the initial enzyme activity, and the relative enzyme activity was only 30% after 2 h, while the relative enzyme activity of immobilized enzyme stored for 2 h was still as high as 94%. With the increase in temperature, the relative enzyme activity of the free enzyme decreased sharply with holding time: the relative enzyme activity was less than 50% after storage at 60 °C for one hour, the relative enzyme activity was only 20% after 2 h, and the immobilized enzyme was more than 90% of the initial enzyme activity after 2 h storage at 60 °C. It can be seen that the PAES-C/P vector can better protect the enzyme from the influence of temperature and that the thermal stability was greatly improved after the enzyme was immobilized.

**Figure 8:**
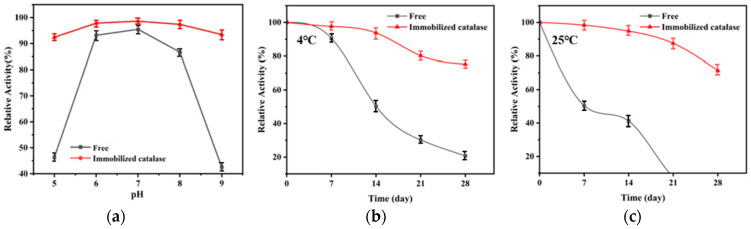
(**a**) Comparison of the pH stability of immobilized enzymes and free enzymes, (**b**) comparison of immobilized enzymes with free enzymes at 4 °C, showing the storage-stability ratio, (**c**) comparison of immobilized enzymes with free enzymes at 25 °C, showing the storage-stability ratio.

It can be seen from Figure 8a that the immobilized enzyme showed better relative enzyme activity than the free enzyme in the pH range investigated, a pH range of 5.0~9.0. The optimal reaction pH of the immobilized enzyme was 7; the optimal pH of the free enzyme was 7; and when the pH = 5, the activity of the immobilized enzyme was 95% and the activity of the free enzyme was 93%. When pH = 9, the relative enzyme activity of the immobilized enzyme was 91%, and the relative enzyme activity of the free enzyme was 91%. It was found that after immobilization of CAT by the PAES-C-doped PVP carrier, the sensitivity to pH decreased and the optimal reaction temperature pH was 7.

The storage stabilities of immobilized and free enzymes were explored at 4 °C and 25 °C, respectively, as shown in Figure 8b,c. The immobilized enzyme obtained from PAES-C/P was stored longer and had higher relative enzyme activity at 4 °C; it retained more than 75% relative enzyme activity on the 28th day, while the relative activity of the free enzyme was only 21% of the initial enzyme activity; at 25 °C, the immobilized enzyme prepared with PAES-C/P retained more than 70% of the initial enzyme activity on the 28th day, while the free enzyme was almost completely inactivated on the 21st day. It was found that at 4 °C, due to the exposure of free enzymes to air, the enzyme activity gradually decreased as storage time increased; at 25 °C, free enzymes were greatly affected by environmental factors and were easily inactivated; after the CAT was immobilized by the PAES-C-doped PVP carrier, the immobilized enzyme had more stable storage stability than free enzyme under low-temperature or room-temperature conditions.

**Figure 9:**
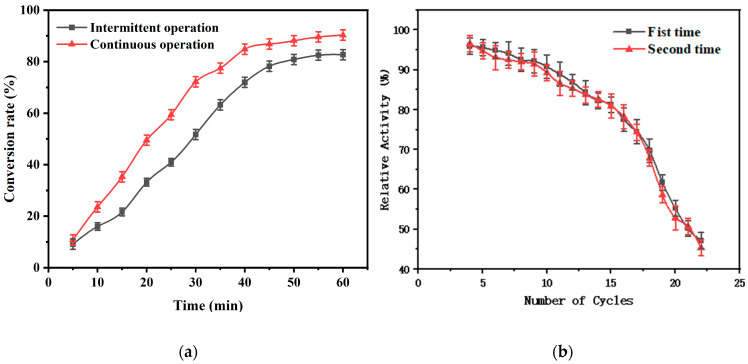
(**a**) Recyclability tests of immobilized enzymes, (**b**) Comparison of continuous and intermittent operations for immobilized enzymes.

The reusability of the immobilized catalase is an important issue for the implementation of a cost-effective enzymatic process. The usability of immobilized enzymes was verified in experiments exploring the operational stability of immobilized enzymes. A certain amount of H_2_O_2_ reacted with the immobilized enzyme at 30 °C for 5 min, and the obtained immobilized-enzyme activity was considered to be 100%. The immobilized enzyme was then filtered out and an equal amount of H_2_O_2_ was added to it, and the results of 22 cycles are shown in Figure 9b. Figure 9b shows that after 22 catalyzed reactions with immobilized enzymes, re-immobilized CAT can still show good recyclability. After 22 consecutive catalytic hydrogen peroxide decomposition reactions, the enzyme activity was about 45% of the initial enzyme activity. In the first 10 reactions, there was no significant decrease, and there was almost no difference from the first reuse, which further indicates that the carrier was not lost and shows good reusability.

The effect of immobilized enzymes on the H_2_O_2_ conversion rate in batch operation and continuous operation was measured with a gradient of 5 min, and the results are shown in Figure 9a. When the reaction time was half an hour, the conversion rate of H_2_O_2_ with immobilized enzyme and intermittent operation was 52% and the conversion rate with continuous operation could reach 72%; with the increase in reaction time, the conversion rate of H_2_O_2_ by the immobilized enzyme with continuous operation (90%) was higher than the conversion rate by the immobilized enzyme with continuous operation (83%) after one hour. Therefore, the continuous operation of H_2_O_2_ decomposition by immobilized enzymes is better than batch operation.

The better stability of the immobilized catalase compared to that of the free catalase in this process can be attributed to the immobilization of the catalase on PAES-C, which can prevent protein–protein aggregation and maintain the molecular conformation of the enzyme. In addition, the separation is relatively mild and easy, which effectively prevents enzyme leakage.

### 3.4. Catalytic Decomposition of H_2_O_2_

**Figure 10:**
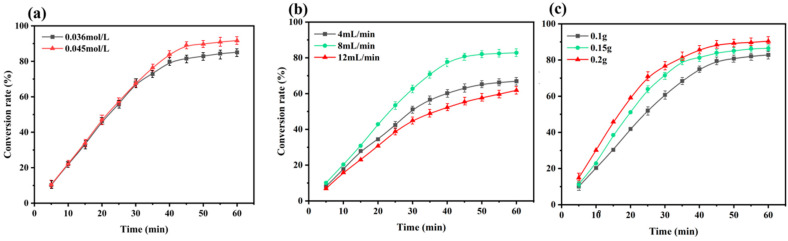
(**a**) Effect of initial concentrations on continuous catalytic conversion of H_2_O_2_; (**b**) effect of solution flow rate on continuous catalytic conversion of H_2_O_2_; (**c**) effect of mass of immobilized enzyme on continuous catalytic conversion of H_2_O_2_.

In the experiment with continuous decomposition of H_2_O_2_ by immobilized enzymes, in order to obtain the best experimental conditions, the conversion rate of H_2_O_2_ was tested against three single factors: initial H_2_O_2_ concentration, solution flow rate and immobilized-enzyme quality. The results are shown in Figure 10a–c: when the substrate concentrations are 0.036 mol/L and 0.045 mol/L, the conversion rate of H_2_O_2_ in the first half hour showed little change and the conversion rate of substrate in the second half of 0.045 mol/L was higher than that at 0.036 mol/L. The conversion rate of H_2_O_2_ first increased and then decreased with an increasing solution flow rate: the conversion rate was 67% when the flow rate was 4 mL/min after a 1 h reaction, and the maximum conversion rate was 83% at 8 mL/min and 62% at 12 mL/min. The conversion rate of H_2_O_2_ also increased with increased carrier mass: when the carrier mass was 0.2 g, and after 1 h of reaction, the H_2_O_2_-conversion rate reached as high as 90%. This result shows that the above three factors have different degrees of influence on the continuous decomposition of H_2_O_2_ by immobilized enzyme; the greater the substrate concentration and the mass of immobilized enzymes, the higher the conversion rate of H_2_O_2_. By contrast, excessive flow rate of the solution will lead to insufficient contact between H_2_O_2_ and immobilized enzymes, resulting in a low conversion rate.

## 4. Conclusions

In this work, PAES-C and cross-linked PVP were combined to prepare a carrier, PAES-C/P, with a suitable pore size. The catalase maintained its initial activity after being successfully loaded onto PAES-C/P, showing good stability and recyclability. With increased temperature, the relative activity of the free enzyme decreased sharply. With increased holding time, the relative enzyme activity was only 20% after 2 h, and the immobilized-enzyme retained more than 90% of the initial enzyme activity after 2 h storage at 60 °C. After 22 consecutive catalytic hydrogen peroxide-decomposition reactions, the enzyme activity was still about 45% of the initial enzyme activity. The enzyme activity in the first 10 reactions showed no significant decrease, and there was almost no difference from the first reuse. In addition, PAES-C/P can be successfully applied to the continuous catalytic decomposition of H_2_O_2_. Based on its porous structure, high mechanical strength, and easy recyclability, PAES-C-immobilized enzymes will have a wide range of applications in industrial processes involving enzymes.

## Figures and Tables

**Table 1:** Preparation schemes for PAES-C/K90 (PAES-C/P).

Type	K90				PVP-P			
Solid content (%)	12	15	18	20	12	15	18	20
DMSO (%)	88	75	82	80	88	75	82	80

**Table 2:** Immobilized enzyme single-factor assay.

Experimental Conditions	Concentration of Enzyme (U/mL)	Temperature (°C)	Time (h)
Concentration of enzyme (U/mL)	16, 32, 48, 64, 80, 96, 112	---	---
Temperature (°C)	---	25, 30, 35, 40, 45, 50	---
Time (h)	---	---	2, 4, 6, 8, 10, 12

**Table 3:** Experimental conditions for catalytic decomposition of H_2_O_2_ by immobilized catalase.

Experimental Conditions	H_2_O_2_ Initial Concentration (mol/L)	Velocity of Flow (mL/min)	Mass of Immobilized Enzyme (g)
H_2_O_2_ initial concentration (mol/L)	0.035, 0.045	---	---
Velocity of flow (mL/min)	---	4, 8, 12	---
Mass of immobilized enzyme (g)	---	---	0.1, 0.15, 0.2

**Table 4:** Effect of different concentrations of Cu^2+^ on immobilized-enzyme activity.

	Activation					Suppression				
Concentration (mmol/L)	1	2	3	4	5	10	20	30	40	50
Relative Activity (%)	125.34	143.58	181.53	152.48	102.60	28.06	23.84	14.71	8.78	5.86

## Data Availability

Data are contained within the article.

## Supplementary Materials

The following supporting information can be downloaded at: https://www.mdpi.com/article/10.3390/polym16131762/s1, Formula (S1): Determination of free catalase (CAT) enzyme activity; Formula (S2): Determination of immobilized catalase (CAT) enzyme activity; Formula (S3): Calculation formula of adsorption rate of immobilized enzyme; Formula (S4): Calculation formula of recovery rate of immobilized enzyme; Figure S1: Hydrogen peroxide standard curve.
