# Supplementary material for: Enhanced Hydrogen Peroxide Decomposition in a Continuous-Flow Reactor over Immobilized Catalase with PAES-C
Source: Polymers (Basel). 2024 Jun 21;16(13):1762. doi: 10.3390/polym16131762 (PMC11243836; doi:10.3390/polym16131762)
Supplement: Supplementary file 1 [file polymers-16-01762-s001.zip › polymers-3031284-supplementary.pdf]

Enhanced hydrogen peroxide decomposition in a continuous flow reactor over immobilized catalase with PAES-C

Yunrui Li [1], Yu Zhang [1] , Wenyu Zhang [2] , Hao Wu [3] and Shaoyin Zhang [*]

School of Light Industry & Chemical Engineering, Dalian Polytechnic University, Dalian 116034, China;
210120856000122@xy.dlpu.edu.cn;
210120856000132@xy.dlpu.edu.cn;
210110703000008@xy.dlpu.edu.cn;
1490453606@qq.com
* Correspondence: zhangsy@dlpu.edu.cn; Tel.: +1-313-044-9492

Determination of Hydrogen peroxide content

UV visible spectrophotometry was used to analyze the content of enzyme Hydrogen peroxide in the samples. Accurately prepare hydrogen peroxide solutions of different concentrations, and measure their absorbance A at their characteristic absorption wavelength of 240 nm. Using solution concentration C (mol/L) as the abscissa and absorbance A as the ordinate, perform linear regression analysis to obtain a standard curve related to solution concentration and absorbance, as shown in Figure S1.

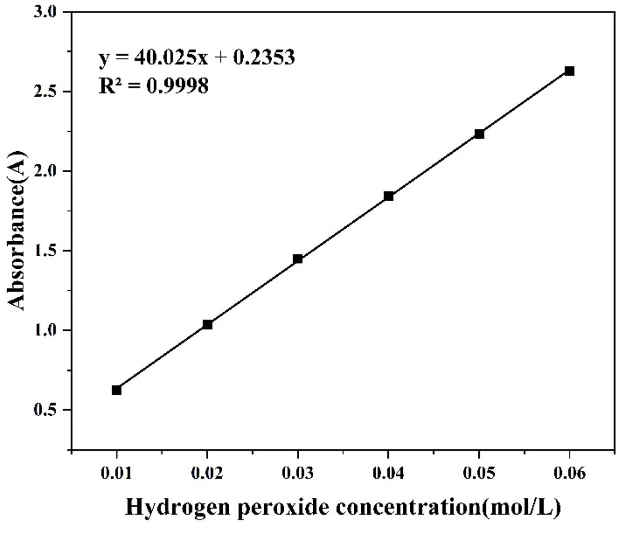

Figure S1. Hydrogen peroxide standard curve

Method for determination of enzyme activity and related formulas

Determination of free catalase (CAT) enzyme activity

2.9 mL 0.1mol/L H_2O_2 solution and 0.1 mL CAT enzyme sample were added into a quartz cup with reaction temperature of 25 °C and buffer pH=7.0. The decomposition rate of H_2O_2 was determined at OD 240nm.

The calculation formula of catalase activity (*U/mL*) is calculated as follows:

$$\text{Free catalase activity } (U/mL) = \frac{V_0 \times n \times 1000 \times \Delta OD_{240}}{\Delta t \times 43.6 \times V_1} \quad \text{(S1)}$$

Where n is the dilution ratio; V_0 is the total volume of the mixture; V_1 is the volume of the enzyme solution of the sample; 43.6mol/Lcm is the molar extinction coefficient of H_2O_2 at OD_{240}.

Determination of immobilized catalase (CAT) enzyme activity

Definition of enzyme activity: The amount of immobilized enzyme required to decompose 1 umoL of hydrogen peroxide per minute at a temperature of 30 °C, pH=7.0 under defined conditions (based on the total mass of the immobilized enzyme). The unit of enzyme activity is expressed in U/mL.

$$\text{Immobilized catalase activity } (U/mL) = \frac{V(C_0 - C_e) \times 1000}{T \times m} \quad \text{(S2)}$$

V is the volume of hydrogen peroxide solution; C_0 is initial concentration of hydrogen peroxide; C_e is hydrogen peroxide equilibrium

concentration; T is reaction time; m is the total mass after immobilization of the enzyme.

Calculation formula of adsorption rate and recovery rate of immobilized enzyme

$$\text{Adsorption rate} = \frac{\text{Total free enzyme} - \text{serum enzyme}}{\text{Total free enzyme}} \times 100\% \quad \text{(S3)}$$

$$\text{Recovery rate} = \frac{Total\ enzyme\ activity\ of\ immobilized\ enzyme}{Total\ enzyme\ activity\ of\ free\ enzymes - Total\ enzyme\ activity\ of\ supernatant} \quad \text{(S4)}$$

Immobilized catalase condition optimization

Optimal amount of enzyme: The amount of carrier was set to 0.05 g, the immobilization temperature was 30 °C, the oscillation speed was 150 r/min, the pH was 7.0, and the immobilization time was 6 h, and carry out the different enzyme concentrations at 16, 32, 48, 64, 80, 96, 112 U/mL respectively. The final result was determined by repeating three times. The immobilized enzyme activity was 100 % relative to the enzyme activity under the optimal condition.

Optimal temperature: The amount of carrier was set to 0.05 g, the oscillation speed was 150 r/min, pH=7.0, the immobilization time was 6 h, the amount of enzyme was 3 mL, and carry out the temperature at 25, 30, 35, 40, 45, 50 °C respectively. The final result was determined by repeating three times. The immobilized enzyme activity was 100 % relative to the enzyme activity under the optimal condition.

Optimal time: The amount of carrier was set to 0.05 g, the oscillation speed was 150 r/min, pH=7.0, the amount of enzyme was 3 mL, the temperature was 30 °C, and perform the time of the immobilized catalase condition at 2, 4, 6, 8, 10, 12 h respectively. The final result was determined by repeating three times. The immobilized enzyme activity was 100 % relative to the enzyme activity under the optimal condition.

Experimental conditions for catalytic decomposition of H_2O_2 by PAES-C/P-CAT

H_2O_2 initial concentration: The velocity of flow was set to 8mL/min, PAES-C/P-CAT 0.2 g (Dry weight), pH=7, and carry out the H_2O_2 initial concentration at 0.036 mol/L and 0.045 mol/L respectively. The reaction was carried out to determine the enzyme activity of the immobilized enzyme and the free enzyme, and the experiment was repeated three times, and the enzyme activity was taken as the highest enzyme activity as 100 % relative enzyme activity.

Velocity of flow: PAES-C/P-CAT was set to 0.15 g (Dry weight), pH=7, the H_2O_2 initial concentration 0.045 mol/L, and the velocity of flow was set to 4, 8, 12 mL/min respectively. The reaction was carried out to determine the enzyme activity of the immobilized enzyme and the free enzyme, and the experiment was repeated three times, and the enzyme activity was taken as the highest enzyme activity as 100 % relative enzyme activity.

Immobilized enzyme mass: The H_2O_2 initial concentration was set to 8

mL/min, pH=7, the H_2O_2 initial concentration 0.045 mol/L, and PAES-C/P-CAT was set to 0.2, 0.4, 0.6 g respectively. The reaction was carried out to determine the enzyme activity of the immobilized enzyme and the free enzyme, and the experiment was repeated three times, and the enzyme activity was taken as the highest enzyme activity as 100 % relative enzyme activity.

Stability of catalase immobilized on PAES-C

Thermal stability: The prepared immobilized enzyme was placed in phosphate buffer solution at pH 7.0 and stored at 40, 50 and 60 °C for 30, 60, 90 and 120 min respectively, and the changes of enzyme activity of immobilized enzyme at different temperatures and different times were calculated according to formulas S2, and the thermal stability of the free enzyme was measured as a control under the same conditions, and the immobilized enzyme activity was 100 % relative to the enzyme activity without heat treatment, and the final result was determined by three replicates.

pH stability: The prepared immobilized enzyme was placed in phosphate buffer solution at different pH (5.0~9.0) and stored at room temperature for 5 h, the changes of enzyme activity of immobilized enzyme in different pH ranges were calculated according to formulas S2, and the pH stability of free enzyme was measured as a control under the same conditions, and the immobilized enzyme activity was 100 % relative to the enzyme activity without acid and base treatment, and the final result was determined by three

repeats.

Storage stability: The prepared immobilized enzymes were placed in air and stored at 4 and 25 °C for 28 days respectively, and their enzyme activities were calculated every 7 days according to formulas S2, and the storage stability of the free enzymes was determined as a control under the same conditions, and the immobilized enzyme activities were determined with the enzyme activity measured for the first time as 100 % relative enzyme activity, and the final result was determined by repeating three times.

Operational stability: The porous carrier adsorbed Cu^{2+} and free enzyme respectively, and the prepared immobilized enzyme was placed in a 30 °C water bath shaker, and the immobilized enzyme activity was calculated according to formulas S2, and the obtained immobilized enzyme activity was 100 % relative to the enzyme activity measured for the first time, and the final result was determined by repeating three times.
